# Interplay between abiotic conditions and mycorrhizal abundance determines differentiation and potential adaptation in a Mediterranean orchid

**DOI:** 10.1093/aob/mcag219

**Published:** 2026-07-23

**Authors:** Marco G Balducci, Karl J Duffy

**Affiliations:** Department of Biology, University of Naples Federico II, Complesso Universitario di Monte Sant’Angelo, Naples 80126, Italy; Department of Biology, University of Naples Federico II, Complesso Universitario di Monte Sant’Angelo, Naples 80126, Italy

**Keywords:** ddRADseq, gene flow, mycorrhizal fungi, population genetics, spatial genetic structure

## Abstract

**Background and Aims:**

Disentangling the relative influence of abiotic and biotic factors on plant population differentiation remains a major challenge in evolutionary ecology yet is essential for understanding the drivers of local adaptation. Orchids are suitable for addressing this question since they often occur in patchily distributed populations, and all orchids depend on orchid mycorrhizal (OrM) fungi for seed germination and establishment. Hence, local abiotic conditions together with OrM fungi may influence population differentiation and adaptation.

**Methods:**

Based on 316 952 polymorphic single nucleotide polymorphisms (SNPs) sampled from 21 populations across the central Mediterranean range of *Orchis italica*, we performed a suite of analyses to test how population differentiation and potential adaptation are influenced by the interplay between abiotic factors and OrM fungi. Outlier detection and functional annotation were performed on a subset of SNPs aligned to the *Ophrys sphegodes* reference genome.

**Results:**

We found strong differentiation at the regional level, while loci under selection were associated with temperature, precipitation regime, soil texture and the abundance of specific OrM fungal taxa. Functional annotation revealed that outlier SNPs were enriched for genes involved in transcription and RNA processing, signal transduction, stress response and metabolic processes, including in the presence of OrM fungi.

**Conclusions:**

Abiotic conditions and OrM fungi jointly determine differentiation in *O. italica*. Identifying the selective pressures underlying differentiation and adaptive variation is critical for understanding plant responses to ongoing environmental change.

## INTRODUCTION

Genetic diversity is frequently partitioned among populations of species, often leading to population differentiation and adaptation to local environments (Givnish, 2010). Abiotic factors, such as climate and soil conditions, play important roles in determining genetic differentiation of populations of species across large geographical ranges (Hawkins *et al.*, 2003; Pearson and Dawson, 2003; Fischer *et al.*, 2013), yet the role of biotic factors on genetic differentiation remains poorly understood (Baselga *et al.*, 2012). Populations of most species encounter diverse abiotic and biotic conditions, yet not all species respond to environmental heterogeneity in the same way (Hartke *et al.*, 2021; Gallegos *et al.*, 2023). For instance, species capable of long-distance dispersal often show weak signals of local adaptation and low genetic differentiation, while species with limited dispersal may adapt to local conditions and persist in environmentally heterogenous landscapes (Lenormand, 2002; Räsänen and Hendry, 2008). It is hence necessary to disentangle the relative contributions of abiotic and biotic variables to better understand how genetic structure and selection vary according to variation in their individual effects.

Genetic differentiation generally results as a consequence of gene flow between populations declining with increasing geographical distance, resulting in patterns of isolation by distance (IBD) (Wright, 1943; Hoskin *et al.*, 2005; Ramírez-Barrera *et al.*, 2019). Under IBD, divergence arises from the combined effects of limited gene flow and genetic drift, resulting in spatial dependency of genetic variation, where geographically proximate populations are more genetically similar than those further apart. However, populations may also be structured by environmental differences, leading to isolation by environment (IBE) (Paz *et al.*, 2015; Mosca *et al.*, 2018). IBE arises when gene flow is constrained by natural selection across environmentally heterogenous landscapes (Wang and Bradburd, 2014; Toczydlowski and Waller, 2019). Mechanisms that result in IBE include selection against immigrants, reduced hybrid fitness or biased rates of dispersal regulated by climatic conditions (Wright, 1943; Kawecki and Ebert, 2004; Wang and Bradburd, 2014). These processes can act independently or in concert to partition genetic variation among populations (Sexton *et al.*, 2014). Whereas IBD results solely from distance, IBE may result from the combined influence of multiple abiotic and biotic factors, which in turn leads to local adaptation (Wang and Bradburd, 2014).

All of the estimated 28 000 orchid species (Chase *et al.*, 2015; Christenhusz and Byng, 2016) produce dust-like seeds that lack endosperm, which probably represents an adaptation to wind dispersal (Arditti and Ghani, 2000; Otero and Flanagan, 2006; Hamrick and Trapnell, 2011). Orchid seeds therefore depend on fungi from a phylogenetically disparate guild, termed orchid mycorrhizal (OrM) fungi, to germinate. Despite their capacity for long-distance dispersal, seed germination is often strongly spatially clustered (Jacquemyn *et al.*, 2007). Orchids frequently form small, patchily distributed populations which could be due to preferences for particular habitats (i.e. abiotic factors) and/or by reliance on particular OrM fungi for seed germination and establishment (McCormick and Jacquemyn, 2014; McCormick *et al.*, 2018; Duffy *et al*., 2019; Phillips *et al.*, 2020). These ecological requirements combined have probably strongly influenced orchid distributions, yet the combined roles of abiotic and biotic factors in shaping differentiation in orchids remain poorly understood.

We investigated factors underlying population diversity and differentiation in the Mediterranean orchid, *Orchis italica* Poir. We used *de novo* double-digest RAD sequencing (ddRADseq) on individuals sampled across the central Mediterranean part of its range (southern Italy and Greece) to: (1) determine patterns of fine- and broad-scale spatial genetic differentiation across populations and regions; (2) determine how climatic and soil conditions, and a key biotic variable, OrM fungal abundance, influence genetic differentiation; (3) identify candidate loci potentially under directional selection; and (4) correlate the signal of these candidate loci to both abiotic and biotic factors.

## MATERIALS AND METHODS

### Study species

*Orchis italica* is a diploid perennial orchid that occurs in small (10–20 flowering individuals, occasionally up to 200 flowering individuals) populations distributed across the Mediterranean Basin, from the Iberian Peninsula and North Africa in the west to Greece, Turkey and the Levant in the east. Each plant produces a single terminal racemose inflorescence bearing between ten and 50 pale pink to white flowers that open acropetally within a few days (Kretzschmar *et al.*, 2007). Flowering occurs from the beginning of March until the end of May. The species is self-compatible and is primarily pollinated by generalist bees, occasionally by butterflies and wasps (Pellegrino *et al.*, 2010, 2021). While metabarcoding of OrM communities revealed a high diversity of fungal taxa (Balducci *et al*., 2025), *in vitro* seed germination experiments showed that this species is primarily associated with the genus *Tulasnella* J.Schröt., particularly members of the *T. calospora* (Boud.) Juel complex and this association is maintained throughout adulthood (Balducci *et al*., 2024).

### Sampling

Leaf tissue was collected from 21 populations of *O. italica*, including 19 populations in southern Italy and two in southern Greece (Table 1). From each population, five adult plants were sampled, with individuals spaced at least 10 m apart to minimize the likelihood of sampling close relatives. In one Italian population (Potenza) and both Greek populations, only two individuals per population were sampled (Table 1). Leaves were dried in silica gel, stored at −20 °C, and later used for DNA extraction. Total genomic DNA was extracted from preserved leaf material using the DNeasy Plant mini kit (Qiagen, Hilden, Germany) according to the manufacturer’s protocol. DNA concentration and purity were verified using a NanoDrop 2000 Spectrophotometer (Thermo Fisher Scientific, Wilmington, DE, USA).

**Table 1.** Summary genetic statistics for all populations for only variant nucleotide positions that are polymorphic in at least one individual. These statistics include the number of leaves and roots collected from each population, the number of variable sites unique to each population (private), the average observed heterozygosity per locus (*H*_o_), the average expected heterozygosity per locus (*H*_e_) the average nucleotide diversity (π) and the average Wright’s inbreeding coefficient (*F*_IS_).

Region	Population	Population ID	Leaves collected	Roots collected	Private alleles	*H*_o_	*H*_e_	π	*F*_IS_
Abruzzo	Majella	MA	5	0	6053	0.06109	0.05157	0.05836	−0.0047
Basilicata	Potenza	PT	2	0	3674	0.05915	0.03175	0.0511	−0.01208
Basilicata	Pollino	PO	5	5	7251	0.06772	0.05608	0.06458	−0.00468
Campania	Anacapri	AN	5	5	73	0.00485	0.00277	0.00524	0.00059
Campania	Matese	MT	5	5	6217	0.05414	0.0509	0.05891	0.01011
Campania	Roccamonfina	RO	5	5	6652	0.05989	0.05319	0.05978	0.0003
Campania	Sorento	SO	5	5	8639	0.05592	0.05979	0.06741	0.0258
Greece	Hymettus	HM	2	2	53	0.00251	0.00126	0.00251	0
Greece	Mount Kyllini	CG	2	2	65	0.00682	0.00348	0.00691	0.00014
Puglia	Bari	BA	5	0	3508	0.05798	0.04453	0.05208	−0.01041
Puglia	Altamura	AT	5	0	5726	0.05947	0.05429	0.06171	0.00556
Puglia	Cagano Varano	CV	5	5	10 313	0.06134	0.06274	0.07049	0.02172
Puglia	Mattinata	MN	5	5	8707	0.05986	0.06333	0.07101	0.02592
Puglia	San Salvatore	SS	5	5	9846	0.06408	0.06402	0.07173	0.01727
Sicily	Gela	GA	5	5	8893	0.06195	0.05419	0.06258	0.0021
Sicily	Santa Ninfa	SN	5	5	9445	0.0608	0.05476	0.06289	0.00472
Sicily	Partanna	PN	5	5	9647	0.06461	0.05647	0.06499	0.00199
Sicily	Ferla	FA	5	5	10 762	0.06287	0.06313	0.07199	0.02021
Sicily	Santa Maria	SM	5	5	11 952	0.0638	0.06292	0.07229	0.01872
Sicily	Santa Caterina Villarmosa	SV	5	5	10 457	0.06401	0.06538	0.0743	0.02338
Sicily	Acate	AE	5	5	14 580	0.06691	0.06733	0.07591	0.02098

### OrM fungal identification

Characterization of the OrM fungal communities associated with *O. italica* across these populations is detailed in Balducci *et al.* (2025); full protocols for root sampling, DNA extraction, ITS amplicon sequencing and bioinformatic processing are provided therein. Briefly, root samples were obtained from the same adult individuals used for genomic analyses in 17 of the 21 populations, and operational taxonomic units (OTUs) were delineated and taxonomically assigned against the UNITE ITS database. In the present study, we used the read abundances of consistently detected fungal OTUs as biotic predictors in subsequent genome–environment association analyses.

### Double-digest RAD sequencing: library preparation and sequencing

Restriction-site associated DNA libraries were sequenced by IGA Technology Services (Udine, Italy) following their custom ddRADseq protocol, with minor modifications to that of Peterson *et al*. (2012). Enzyme combinations were chosen based on the estimated genome size of *O. italica* (8.7 Gbp; Bou Dagher-Kharrat *et al*., 2013). Genomic DNA was fluorometrically quantified, normalized to a uniform concentration, and 300 ng per sample was digested with 2.4 U each of SphI and EcoRI (New England BioLabs) in a 30-μL reaction with CutSmart buffer, incubated at 37 °C for 90 min, followed by 65 °C for 20 min. Digested fragments were ligated to barcoded adapters (2.5 pmol) using 160 U of T4 DNA ligase (New England BioLabs) in a 50-μL reaction, incubated at 23 °C for 60 min, 20 °C for 60 min and 65 °C for 20 min. Samples were multiplexed, purified with 1.5 volumes of AMPure XP beads (Agencourt), and size-selected on a BluePippin instrument (Sage Science) to retain fragments between 440 and 700 bp. Size-selected DNA was amplified using indexed primers and Phusion High-Fidelity PCR Master Mix (New England BioLabs) under the following thermal profile: 95 °C for 3 min; ten cycles of 95 °C for 30 s, 60 °C for 30 s and 72 °C for 45 s; and a final extension at 72 °C for 2 min. Amplified products were purified with AMPure XP beads (Beckman Coulter), and quantified with a Qubit 2.0 Fluorometer (Invitrogen) and Bioanalyzer DNA assay (Agilent Technologies). Sequencing was performed on an Illumina NovaSeq 6000 platform using 150-bp paired-end reads, according to the manufacturer’s instructions (Illumina, San Diego, CA, USA).

### Quality filtering and variant calling

Raw reads were processed based on the Stacks software suite (Catchen *et al*., 2013). Demultiplexing of Illumina reads was performed with process_radtags (Catchen *et al*., 2013), followed by read assembly for each sample with ustacks. A catalogue of loci was generated with cstacks, and individual samples were matched against this catalogue using sstacks and tsv2bam. The gstacks module was then used to assemble paired-end contigs, merge them with single-end loci, align reads to loci and call single nucleotide polymorphisms (SNPs). Loci were filtered with the ‘population’ program in Stacks v.2.53 (Catchen *et al*., 2013), retaining only loci present in at least 75 % of individuals across all samples (−R 0.75). To reduce the risk of paralogous sequences, loci with observed heterozygosity greater than 0.8 (−max-obs-het 0.8) were excluded.

### Genetic diversity and population differentiation

All analyses were performed in R v.4.0 (R Core Team, 2026) unless otherwise stated. Genetic diversity and differentiation statistics were calculated using the ‘populations’ program in Stacks (Catchen *et al*., 2013). For each population, we estimated observed heterozygosity (*H*_o_), expected heterozygosity (*H*_e_), nucleotide diversity (π) and inbreeding coefficients (*F*_IS_). Pairwise population differentiation was assessed using *F*_ST_.

We inferred individual ancestry coefficients and evaluated population structure using the sparse non-negative matrix factorization (sNMF) algorithm implemented in the R package ‘LEA’ v.2.8.0 (Frichot *et al*., 2014; Frichot and François, 2015). Ten values of *K* (*K* = 2–10) were tested with 20 iterations each, and the number of ancestral populations was evaluated using the cross-entropy criterion. Since structure-like methods may underestimate or misidentify *K* (Kalinowski, 2011; Lawson *et al.*, 2018), we explored the full range of *K* values to assess potential hierarchical structure. We tested for population structure using discriminative analysis of principal components (DAPC) (Jombart *et al.*, 2010) in the R package ‘adegenet’. Cross-validation with 1000 permutations was used to determine the number of principal components retained. *K*-means clustering solutions were compared using the Bayesian information criterion (BIC), and individuals were assigned to populations based on the retention of 60 % of cumulative variance.

To complement clustering approaches, we reconstructed phylogenomic relationships using IQ-TREE v.2.0.3 (Nguyen *et al*., 2015). Model selection was performed with ModelFinder Plus (-m MFP), including ascertainment bias correction (ASC; Lewis and Puterman, 2001). Nodal support was assessed with 1000 ultrafast bootstrap replicates (-bb 1000). The resulting unrooted phylogeny was visualized and annotated using the Interactive Tree of Life (https://itol.embl.de/).

### Genetic variation associated with spatial, abiotic and biotic factors

To test for IBD and IBE, we compared pairwise population genetic distances (Nei’s *D*) with geographical and environmental distances (see environmental data below) using Mantel tests. We used the ‘mantel’ function from the R package ‘vegan’ (Oksanen *et al.*, 2019), with Spearman rank correlation and 9999 permutations. Geographical distances among populations were calculated as Haversine distances using the ‘distm’ function in the ‘geosphere’ package (Hijmans *et al.*, 2017). Environmental distances were calculated as Euclidean distances using the ‘dist’ function.

Partial redundancy analysis (pRDA) was used to quantify the relative contributions of spatial distance, environmental predictors and biotic interactions in explaining adaptive genetic variation across populations (Capblancq and Forester, 2021). This approach is well suited to genomic datasets as it does not rely on population grouping assumptions or equilibrium requirements inherent to *F*_ST_-based analyses. Spatial autocorrelation was addressed by converting pairwise geographical distances to principal coordinates of neighbour matrices (PCNMs) using the ‘pcnm’ function, with significant spatial eigenvectors (PCNM 1 and PCNM 2) included as conditional variables. Environmental predictors included 19 WorldClim bioclimatic variables (1950–2000; Fick and Hijmans, 2017) and soil parameters from the LUCAS Database (ESDAC; Van Liedekerke *et al.*, 2006; Ballabio *et al.*, 2016), at 1-km resolution in Quantum GIS (QGIS Development Team, 2024). All variables were log-transformed to normalize scales. Forward selection (‘forward.sel’ function in ‘vegan’) with 9999 permutations identified optimal predictors, while variance inflation factors (VIFs) using the ‘vif.cca’ function controlled for multicollinearity among bioclimatic and soil variables. The ‘envfit’ function assessed predictor significance in explaining genetic variation.

For 17 of the 21 populations, we included abundance, as measured by the number of reads, of six OrM fungal OTUs that were consistently detected in all adult individuals of *O. italica* in our previous community-level characterization (see Balducci *et al.*, 2025). Among these, tul26, tul27, tul28 (Tulasnellaceae) and cer15 (Ceratobasidiaceae) were the most abundant and consistently present OTUs detected across populations, making them ideal candidates for inclusion in explanatory models. Population allele frequencies were used as the response matrix, with three classes of predictors: (1) environmental, (2) geographical (PCNM as conditional variables) and (3) OrM fungal abundances. Forward selection retained three key environmental variables: bio1 (annual mean temperature), bio17 (precipitation in the driest quarter) and coarse fragments. Coarse fragments are used here as a proxy for soil drainage capacity, a habitat-scale property known to influence the persistence of Mediterranean orchids and their associated OrM fungal communities (McCormick and Jacquemyn, 2014; Vogt-Schilb *et al*., 2020), rather than as a direct measure of root–substrate interaction. We implemented four pRDA models using the ‘rda’ function in the ‘vegan’ package (Oksanen *et al.*, 2019): (1) abiotic-only (all 21 populations to maximize genomic coverage); and three further models run on the 17-population subset for which OrM fungal data were available, allowing direct comparison on the same dataset: (2) abiotic-only; (3) OrM fungi only; and (4) combined abiotic–biotic. Model significance, axis contributions and variable effects were assessed using permutation-based analysis of variance (ANOVA; 999 iterations).

### Putative loci under selection

To identify SNPs putatively under natural selection, we implemented a dual approach combining population-differentiation tests and environmental association analyses (de Villemereuil *et al.*, 2014). For population differentiation, we used pcadapt (Luu *et al.*, 2017) at a Bonferroni-corrected significance of α = 0.05, and OutFLANK (Whitlock and Lotterhos, 2015), which calculates *F*_ST_ based on the 21 sampled populations and is robust to false positives. OutFLANK was run with default parameters and a *q-*value threshold of 0.1, corresponding to a 10 % false discovery rate (Benjamini and Hochberg, 1995).

For environmental associations, we used pRDA on SNP loadings from the first three constrained axes (Forester *et al*., 2018). Candidate outliers were identified as SNPs exceeding 3.5 standard deviations from the mean loading (*P* < 0.0005). When loci were associated with multiple RDA axes, Pearson’s correlation was used to assign the strongest predictor.

Variant calling and outlier detection were performed in the coordinate system of the chromosome-scale genome of *Ophrys sphegodes* (Russo *et al*., 2024), a closely related orchid in the same subtribe (Orchidinae); no chromosome-scale annotated nuclear genome is currently available for *Orchis*. Trimmed reads from all 96 individuals were aligned to the *O. sphegodes* reference with BWA-MEM v.0.7.19 (Li and Durbin, 2009), de-duplicated with samtools markdup, and variants jointly called with bcftools mpileup + call (MAPQ ≥ 20, base quality ≥ 20). Biallelic SNPs were retained after applying a quality filter (QUAL ≥ 20, F_MISSING ≤ 0.5, MAF ≥ 0.05), yielding 15 707 SNPs that formed the analysis set. Outlier SNP positions were intersected with the high-quality protein-coding gene annotation of Russo *et al*. (2024) using BEDTools v.2.31.0 (Quinlan, 2014). Functional annotations, including Gene Ontology terms, InterPro domains and KEGG pathways, were obtained from the functional annotation table of Russo *et al*. (2024).

## RESULTS

### Population diversity and differentiation

After quality filtering to remove loci with high missing data, excessive heterozygosity and low coverage, we retained 316 952 genotyped SNPs from 96 individuals representing 21 populations (Table 1). Italian populations (excluding Anacapri) showed consistently high levels of genetic diversity, with nucleotide diversity (π) ranging from 0.051 to 0.076, observed heterozygosity (*H*_o_) from 0.054 to 0.068, and expected heterozygosity (*H*_e_) from 0.032 to 0.067 (Table 1). In contrast, the two Greek populations and Anacapri showed reduced diversity across all metrics (*P* < 0.005, Table 1). Private alleles were detected in all populations, ranging from 53 in Hymettus (Greece) to 14 580 in Acate (Sicily). The inbreeding coefficient (*F*_IS_) was uniformly low across populations (range: −0.012 to +0.026; Table 1) and was slightly positive in 17 of 21 populations. Pairwise *F*_ST_ values ranged from 0.064 to 1 (Supplementary Data Fig. S1), and all comparisons were significantly greater than zero. The strongest apparent differentiation was observed in the Greek populations, with pairwise *F*_ST_ values approaching 1 in comparison with Italian populations.

### Association of genomic variation with geography and environment

There was a positive correlation between geographical and genetic distances, supporting the IBD hypothesis (*r* = 0.347, *P* = 0.01; Supplementary Data Fig. S2). In contrast, based on the raw genetic distance matrix, the IBE hypothesis was not supported, with no association between environmental and genetic distances (*r* = 0.239, *P* = 0.407; Supplementary Data Fig. S3).

As reported in Balducci *et al.* (2025), the read abundance of these consistently detected OTUs varied substantially across populations along longitudinal and latitudinal gradients. Specifically, the abundance of tul26, tul27, tul28 and cer15 increased with increasing longitude and decreased with increasing latitude, indicating that overall OrM fungal abundance is highest in southern and eastern populations of the sampled range. We used the per-population read abundance of these four OTUs as biotic predictors in the pRDA models described below.

The pRDAs controlling for geographical distance revealed that both abiotic and OrM communities significantly influenced genetic structure. Across the full dataset, abiotic predictors explained a significant proportion of genetic variation (*P* = 0.001, Fig. 1A), while in the 17-population subset the effect was weaker, yet still significant (*P* = 0.045, Fig. 1B). OrM community composition alone had a strong effect (*P* = 0.001, Fig. 1C), with the combined abiotic + OrM fungal model providing the best fit overall (*P* = 0.005, Fig. 1D).

**Fig. 1.**
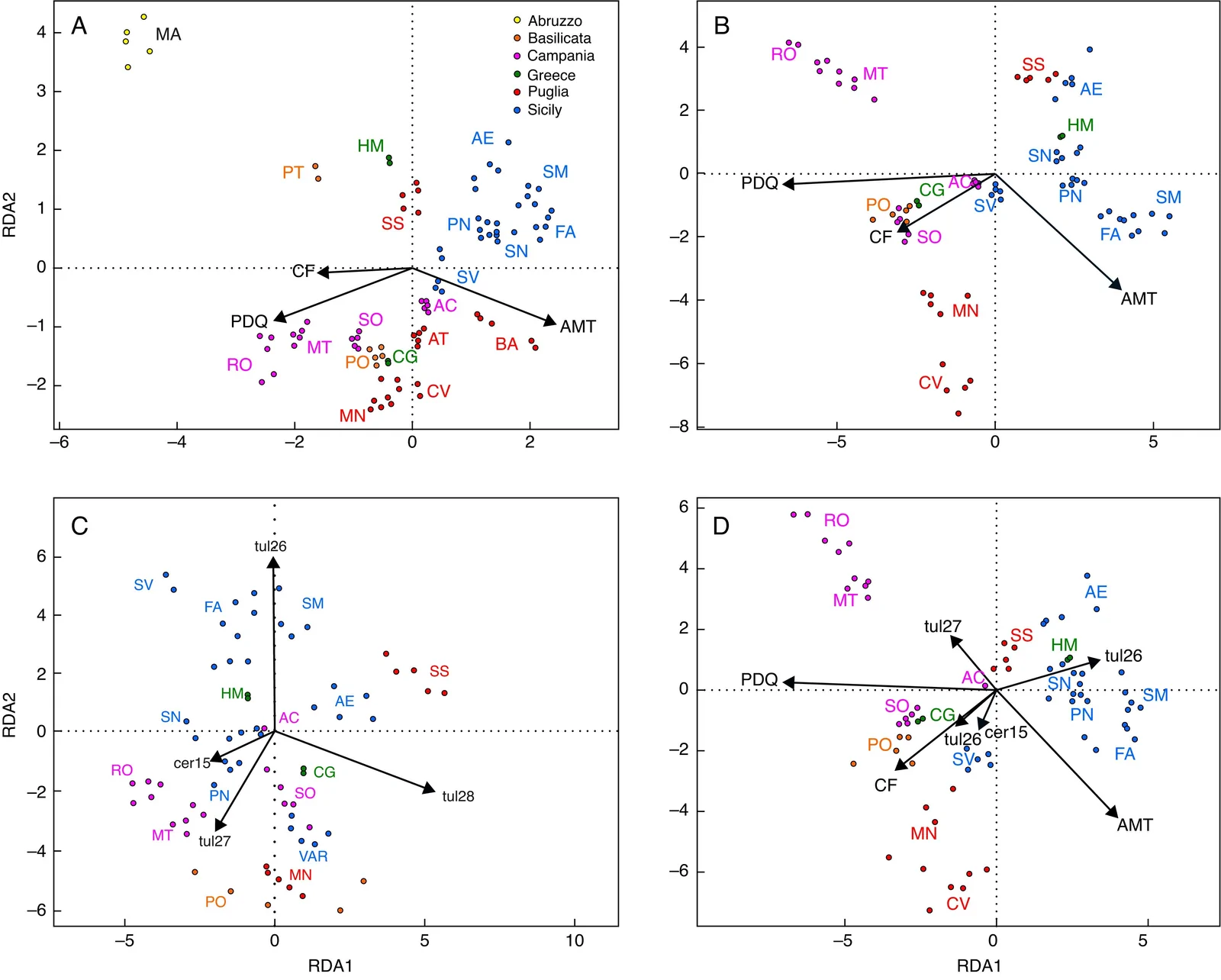
Partial RDAs of *Orchis italica* allele frequencies. Panels show the effects of (A) abiotic predictors on all 21 populations, (B) abiotic predictors on the 17-population subset with fungal data, (C) mycorrhizal community predictors, and (D) combined abiotic and biotic predictors. Abiotic predictors: coarse fragments (CF), precipitation of the driest quarter (PDQ), annual mean temperature (AMT). Biotic predictors: tul26, tul27, tul28, cer15.

The full population analysis showed distinct genetic clustering in central and southern Italy (particularly Majella), with individuals from Roccamonfina and Matese diverging along a precipitation gradient (Fig. 1A). In the 17-population subset, genetic variation in Pollino (Basilicata), Hymettus (Greece) and Sorrento (Campania) was strongly associated with soil drainage (coarse fragments; Fig. 1B). In Sicily, populations from Santa Caterina Villarmosa, Ferla and Santa Maria were positively associated with tul26, while Santa Ninfa and Partanna showed negative associations with cer15 (Fig. 1C). Northern Italian populations, along with Roccamonfina and Matese, were negatively associated with tul27 (Fig. 1C). The combined model explained the greatest variation, identifying tul26 as the primary driver for Sicilian populations, coarse fragments for central Italian populations (Pollino and Sorrento) and cer15 for Santa Caterina Villarmosa (Sicily; Fig. 1D).

Across the five detection methods, we identified 194 unique candidate SNPs putatively under selection (Supplementary Data Table S1). pcadapt identified 54 outliers and OutFLANK identified 106 outliers based on all 96 individuals across 21 populations. The 21-population pRDA (abiotic predictors only) detected 25 outlier SNPs, with coarse fragments emerging as the strongest predictor (14 SNPs), followed by precipitation of the driest quarter (six SNPs) and annual mean temperature (five SNPs). The three pRDAs of the 17-population subset (*n* = 79 individuals) detected 22 outliers in the abiotic-only model, 21 in the OrM-only model and 13 in the combined abiotic + biotic model. In the 17-population abiotic-only model, most outliers were strongly correlated with coarse fragments (14 SNPs), followed by precipitation of the driest quarter (seven) and annual mean temperature (one). The OrM-only model identified 13 SNPs linked to tul26, four to cer15, three to tul28 and one to tul27. The combined model detected outliers most strongly associated with precipitation of the driest quarter (11 SNPs) and coarse fragments (two SNPs). The predictor distribution of outliers per pRDA is summarized in Fig, S4. Thirty-nine SNPs were supported by two or more detection methods, and four by three methods (Table S1).

### Population structure

DAPC identified *K* = 7 as the most likely number of clusters based on the BIC and find.clusters() function. Populations generally grouped together irrespective of geographical distance, except for Majella (Abruzzo, central Italy), which formed a distinct cluster (Supplementary Data Fig. S5). sNMF analysis indicated an optimal *K* = 5, but closer inspection showed that *K* = 3 best captured the major genetic clusters in *O. italica*, as higher values of *K* reflected substructure rather than geographically meaningful population groups. Admixture analysis at *K* = 3 revealed three primary ancestral components: (1) Majella, (2) Sicily, and (3) mainland Italian and Greek populations (Fig. 2A). Additional substructure emerged at higher *K* values. Maximum likelihood phylogenetic analysis, using the best-fit K3Pu + F + R6 model (selected by BIC), recovered strong geographical clustering by region, consistent with admixture results (Fig. 2B). Principal component analysis based on putative outlier SNPs further supported this structure, with populations separating along the first two axes consistent with regional clustering, particularly distinguishing Abruzzo and Sicilian populations (Fig. 2C).

**Fig. 2.**
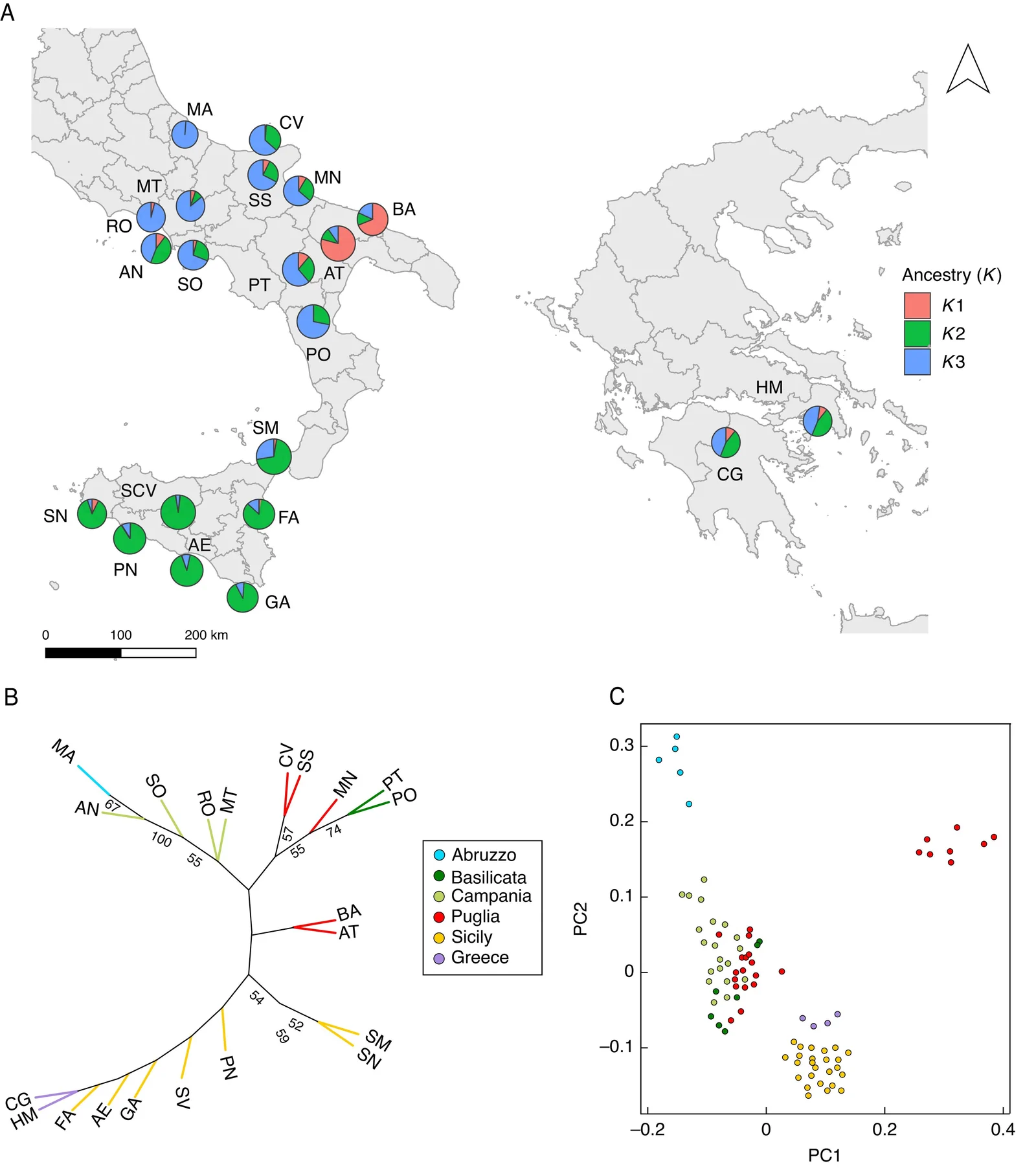
(A) Admixture plots obtained from sNMF analysis showing hierarchical population structure of *Orchis italica* across different *K* values. Population names are abbreviated as in Table 1. (B) Unrooted maximum likelihood tree for 21 populations of *O. italica* from southern Italy and Greece. Each tip represents an individual population which is colour coded to its region. Branch labels indicate support from 10 000 ultrafast bootstrap replicates. (C) Genetic variation based on putative outlier SNPs among populations of *O. italica* along the first two axes of a principal component analysis.

### Putative loci under selection

Of the 194 unique outlier SNPs, 143 (74 %) fell within annotated genes of the *O. sphegodes* reference and 57 in intergenic regions (Supplementary Data Table S2). The 143 genic SNPs corresponded to 90 unique genes, of which 67 (74 %) had at least one functional annotation (Gene Ontology term, InterPro domain or KEGG entry) in Russo *et al*. (2024).

Of the 143 genic outlier SNPs, the majority fell within genes involved in transcription and post-transcriptional regulation (21 SNPs in 12 genes, dominated by pentatricopeptide-repeat RNA-binding proteins), signal transduction and protein-interaction scaffolding (15 SNPs in 11 genes, including WD40-repeat scaffolds, serine/threonine protein kinases and a MORC-family histidine kinase), primary metabolism (12 SNPs in ten genes), stress, defence and chaperone response (11 SNPs in nine genes, including an NB-ARC NLR-type immune receptor, two DnaJ chaperone paralogues and a Clp AAA + ATPase), membrane and organellar transport (11 SNPs in six genes), chromatin and epigenetic regulation (five SNPs in four genes), and DNA repair and recombination (four SNPs in three genes) (Supplementary Data Fig. S6, Tables S3 and S4). Per-category counts of unique genes and SNP gene-pairs are summarized in Table S4. The remaining 62 SNPs fell in 33 genes that were either unannotated (47 SNPs in 23 genes) or assigned only to broader functional categories (15 SNPs in ten genes), and two SNPs corresponded to single hits on a plastid translocon component (TIC214) and a transposable-element-derived locus. Several genes contained multiple outlier SNPs [one pentatricopeptide-repeat (PPR) gene contained ten SNPs; three further genes contained four to six SNPs each], indicating clustered variation in linkage within these loci. Detection methods showed distinct functional category profiles (Fig. S6A–F): pcadapt and OutFLANK detected SNPs in transcription/RNA processing genes, whereas the pRDA models of the 17-population subset showed broader profiles with notable contributions from stress/defence and signalling categories, and the OrM-only model was distinctive in spreading detections across transport, transcription/RNA and stress/defence categories.

## DISCUSSION

The interplay between abiotic and biotic variables influences population differentiation and potential adaptation to local environments. Our results indicate that *O. italica* shows regional genetic differentiation, which probably reflects a combination of historical isolation and contemporary barriers to gene flow. Patterns of differentiation varied with the scale of analysis. While sNMF admixture at *K* = 3 grouped Greek and mainland Italian populations within a shared broad ancestral component, potentially reflecting post-glacial recolonization from eastern refugia (Médail and Diadema, 2009), pairwise *F*_ST_ values and phylogenetic reconstruction revealed apparent contemporary differentiation between Greek and Italian populations at the population and locus level. While most Italian populations have relatively high genetic diversity (as estimated by *H*_o_, *H*_e_ and *π*), the reduced diversity in the Greek populations may reflect long-term demographic processes, although this pattern should be interpreted with caution given the small sample size. Reduced diversity at a sampled site does not always indicate marginal range position: in other orchid species, diversity at range edges has been found to be comparable to that of range centres (e.g. Duffy *et al*., 2009), and pronounced variation can occur between populations even at very local scales (Duffy *et al*., 2020).

The distribution of private alleles provided further insight into differentiation. Eastern Sicilian populations (Acate, Ferla and Gela) harboured elevated numbers of private alleles. One possible explanation is long-term persistence in eastern Sicilian refugia, particularly within the Hyblaean and Erei plateaus, which have been identified among the Mediterranean refugia that allowed plant populations to persist and accumulate distinctive variation across glacial–interglacial cycles (Médail and Diadema, 2009; Feliner, 2014). Eastern Sicily also encompasses ecologically distinct landscapes including volcanic substrates around Mt Etna, with climatic and edaphic conditions that differ from those of western Sicily; such heterogeneity may further promote local selection. In contrast, the Italian island population of Anacapri contained relatively few private alleles, suggesting recent colonization events, limited time for divergence or ongoing gene flow with mainland populations. The two Greek populations (Hymettus and Mount Kyllini) also showed low private allele counts, but this pattern is probably driven by their smaller sample size (*n* = 2 individuals per population) rather than a biological signal.

Despite regional variation in diversity and allele composition, we found consistently low *F*_IS_ values across populations, suggesting that inbreeding is not a major contemporary process shaping genetic variation in *O. italica*. This lack of strong inbreeding is probably maintained by a combination of life-history traits. First, as terrestrial orchids are typically long-lived perennials with extended generation overlap (Tremblay *et al.,* 2005), demographic buffering may reduce the stochastic effects of drift. Second, pollinator-mediated outcrossing due to deceptive pollination characteristic of *Orchis* has wider genetic implications: pollinator learning reduces within-population revisitation, promotes longer-distance pollen movement and enhances gene flow among populations, helping to maintain heterozygosity in fragmented Mediterranean landscapes (Neiland and Wilcock, 1998; Cozzolino and Widmer, 2005; Minasiewicz *et al*., 2018). Together, these features contribute to the maintenance of genetic variation despite the fragmented and patchy distribution typical of Mediterranean orchid populations.

Our analyses revealed that geographically proximate populations of *O. italica* are genetically similar, consistent with IBD and/or shared local adaptation to similar environmental conditions. This indicates that genome-wide neutral genetic structure is primarily shaped by geographical distance. This pattern mirrors findings from a wide range of plant taxa, in which spatial separation and physical barriers dominate genome-wide differentiation, even across environmentally heterogenous landscapes (Holderegger *et al*., 2010; Sexton *et al*., 2014; Mosca *et al*., 2018). Limited dispersal and historical connectivity structures neutral variation, while environmental gradients primarily affect a subset of loci under selection. Our pRDA analyses indicate that particularly in combination with OrM fungal OTU read abundance, climate (temperature and precipitation) and soil properties are putative drivers of adaptive differentiation. While our Mantel-based IBE test was not significant, this probably reflects the limited environmental range of our central Mediterranean sampling, combined with the conservative nature of global Mantel tests when environmental effects are concentrated at specific loci. The pRDAs nonetheless detected significant environmental structuring, indicating that locus-specific environmental selection is still present in *O. italica* despite the absence of a genome-wide IBE signal. Comparable patterns have been observed in alpine and Mediterranean plants, where IBD governs neutral background structure but local adaptation to fine-scale environmental heterogeneity drives selection on specific genomic regions (De Kort *et al*., 2014; Frei *et al*., 2014; Capblancq *et al*., 2020). This distinction between neutral and selective processes has important implications for predicting the capacity of *O. italica* to persist under climate change. Neutral genetic differentiation shaped by IBD suggests that dispersal limitation will constrain natural range shifts, as seen in other orchids with restricted seedling recruitment away from adult populations (Jacquemyn *et al*., 2012). At the same time, the environmental associations of candidate loci imply that local adaptation may depend on narrow climatic thresholds and soil properties, mirroring findings in other species where adaptive genomic variation tracks microenvironmental gradients (Jia *et al*., 2020; Gugger *et al*., 2021). Together, these results highlight the dual role of geography and environment in shaping the evolutionary trajectory of *O. italica* – geographical distance restricts neutral connectivity, while selection linked to environmental heterogeneity and OrM fungi drive adaptive divergence.

Differentiation was strongly associated with abiotic variables such as temperature, precipitation and soil conditions. The Mediterranean is characterized by dry, hot summers and cold, comparatively wet winters (Cowling *et al.*, 2015). Precipitation regimes may impose strong constraints on plants and can act as an important evolutionary driver for adaptation to both drought conditions in the summer and inundation in the winter (Galmés *et al.*, 2007). For *O. italica*, plants typically grow in open grasslands on well-drained calcareous soils, and hence summer droughts, which coincide with early onset of seed germination and development, may impact recruitment. Precipitation regimes, along with higher summer temperatures, may also shape the composition of OrM communities associated with *O. italica* (Balducci *et al.*, 2025).

Our results provide genome-scale evidence that OrM fungi may contribute directly to local adaptation. By integrating SNP markers with fungal abundance patterns, we showed that specific OrM fungal taxa are linked to genetic structure in *O. italica*, potentially acting as biotic filters that influence patterns of adaptation. This was particularly evident in the Sicilian populations, where strong associations with tul26 and cer15 suggest that these OrM fungal OTUs exert variable selective pressures. Such patterns imply that OrM associations can mediate local adaptation to particular ecological conditions, consistent with findings from other orchids where shifts in fungal partners correspond to population differentiation and recruitment success (Jacquemyn *et al*., 2011; Těšitelová *et al*., 2013). Our results therefore suggest that OrM fungi are not mere passive facilitators of orchid seed germination, and highlight that they may act as active drivers of divergence. Indeed, several of the mapped outlier loci were associated with genes involved in cell signalling (including WD40/WW-domain scaffolds, two active serine/threonine protein kinases, a MORC-family histidine kinase and a calcium-dependent C2-domain regulator), PPR RNA processing, chaperone-mediated proteostasis (two DnaJ paralogues, Clp ATPase), chromatin-level transcriptional regulation (including SAGA, SWR1 and SET-domain methyltransferase complexes) and plant innate immunity (an NB-ARC NLR-type receptor), all functions consistent with plant–microbe interactions and host immune/symbiotic response (Parniske, 2008; Bonfante and Genre, 2010; Smith and Read, 2010). Genes linked to chaperone-mediated proteostasis, RNA editing and NLR-mediated recognition are particularly relevant given their roles in nutrient exchange and cellular reorganization during symbiosis (Bago *et al*., 2000; Genre *et al*., 2012), and the recurrent detection of signalling-related genes and chromatin regulators reflects the molecular coordination required to maintain mutualistic balance (Kiers *et al*., 2011; Wang *et al*., 2017).

Population structure analyses further supported these patterns. Both DAPC and sNMF revealed consistent clustering, with individuals from Abruzzo forming a distinct genetic group. This pronounced differentiation probably reflects the region’s complex topography with elevations ranging from ca. 500 m to over 2700 m within short horizontal distances and correspondingly rapid changes in temperature and precipitation. Such topographic and climatic heterogeneity may act as barriers to gene flow by limiting both pollinator movement and seed dispersal (Steinbauer *et al*., 2016; Wu *et al*., 2020). Sicilian populations also showed a distinct genetic profile, although weaker than the divergence observed in Abruzzo. In contrast, high connectivity was observed among populations from Basilicata, Campania, and Puglia, suggesting either historical gene flow or ongoing long-distance dispersal. Nevertheless, even within these well-connected regions, we detected localized differentiation, such as between the geographically proximate populations of Altamura and Bari in Puglia. This fine-scale structure may reflect independent colonization events from distinct founder populations or subsequent reproductive isolation despite close geographical proximity.

Several additional factors are likely to shape population persistence and genetic structure in *O. italica*. First, the species typically occurs in open Mediterranean grasslands and garrigue habitats, where vegetation structure, density and successional dynamics are influenced by fire regimes, grazing and land-use history (Cowling *et al*., 2015), all of which may affect local population sizes, demographic stability, and the abundance and composition of OrM fungi (Vogt-Schilb *et al*., 2020). Second, while our sampling captures substantial variation across the central Mediterranean, *O. italica* extends much further, from the Iberian Peninsula across North Africa to the Aegean and Levant. Populations from unsampled regions may harbour additional genetic variation reflecting deeper phylogeographical structure shaped by Pleistocene refugia (Médail and Diadema, 2009; Feliner, 2014). Although the dust-like seeds of *O. italica* have the potential for long-distance dispersal that may homogenize genetic variation across the core range, peripheral populations may show stronger differentiation and reduced diversity than those reported here. These populations may also associate with distinct OrM fungal communities, given that OrM composition can shift across biogeographical boundaries and climatic gradients (Duffy *et al*., 2019; Balducci *et al*., 2025). Targeted studies integrating fire history, vegetation and land-use data with genomic and mycorrhizal sampling across the full distribution would clarify whether the abiotic–biotic interplay identified here represents a broader feature of *O. italica* or is specific to its central Mediterranean populations, and would test whether OrM fungal taxa critical for germination (see Balducci *et al*., 2024) are consistently associated with the species across its entire range.

## CONCLUSIONS

While geographical distance between populations primarily structures neutral genomic variation, differentiation is strongly influenced by the combination of abiotic variables and abundance of OrM fungi. Such mycorrhizal fungi may act as selective agents shaping population structure in *O. italica*, highlighting the broader role of biotic interactions in plant evolution. Hence, adaptation is not solely a function of climate and soil but also depends on the ecological distribution of compatible fungal partners. Recognizing OrM fungi as potential co-drivers of local adaptation should focus orchid conservation strategies on protecting not just plants but also the below-ground ecological networks that sustain them.

## Supplementary Material

mcag219_Supplementary_Data

## Data Availability

Raw ddRAD sequencing data have been deposited in the NCBI Sequence Read Archive under BioProject accession PRJNA1427967. Individual run accessions are SRR37357204–SRR37357298. Processed data files Processed VCF files and sample metadata are available from the corresponding author upon reasonable request.

## SUPPLEMENTARY DATA

Supplementary data are available at *Annals of Botany* online and consist of the following. Table S1: Candidate outlier SNPs identified across five detection methods in *Orchis italica*. One row per unique outlier SNP (*n* = 194), with genomic position in the *Ophrys sphegodes* reference (Russo *et al*., 2024). Table S2: Per-SNP functional annotation of genic outliers in *Orchis italica*. One row per genic outlier SNP–gene pair (*n* = 143), with SNP genomic position in the *Ophrys sphegodes* reference (Russo *et al*., 2024), the *Ophrys* gene ID, assigned functional category, InterPro domain descriptions, top ten Gene Ontology terms, KEGG description and symbol, EC number and transcription-factor family (where annotated), and method-detection flags for each of the five outlier detection methods. Table S3: Per-gene summary of functional annotation for genic outliers in *Orchis italica*. One row per unique outlier gene in the *Ophrys sphegodes* reference (*n* = 90), with the assigned functional category, full InterPro and Gene Ontology annotation, KEGG description and symbol, and the number of distinct outlier SNPs falling within each gene. Annotations are from Russo *et al*. (2024). Table S4: Functional category counts for *Orchis italica* outlier SNPs and genes. Per-category counts of unique outlier genes and SNP–gene pairs. Categories were assigned by hand-curated keyword matching against InterPro domains and Gene Ontology descriptions in the functional annotation table of Russo *et al*. (2024). Figure S1: Heat map of pairwise *F*_ST_ values for 21 populations of *O. italica*. Deeper colours indicate higher genome-wide differentiation between populations, according to the colour key. Population names are abbreviated as in Table 1. Figure S2: Positive isolation-by-distance relationship (Mantel test: *r* = 0.347, *P* = 0.01) between genetic and geographical distance across populations of *Orchis italica*. Figure S3: Relationship between genetic distance and environmental distance across populations of *Orchis italica*. Figure S4: Predictor distribution of outlier SNPs detected by each partial RDA model. Bars show the number of outlier SNPs whose strongest environmental or biotic correlation matches each predictor, for: (a) the 21-population pRDA based on abiotic predictors only (*n* = 25 outliers); (b) the 17-population pRDA based on abiotic predictors only (*n* = 22); (c) the 17-population pRDA based on orchid mycorrhizal (OrM) community predictors only (*n* = 21); and (d) the 17-population combined pRDA incorporating both abiotic and OrM predictors (*n* = 13). Abiotic predictors: coarse fragments, mean annual temperature, precipitation of the driest quarter. OrM predictors: tul26, tul27, tul28, cer15. Outliers were detected at *z* > 3.5 s.d. from the mean RDA loading on the first three constrained axes. Figure S5: Scatterplots resulting from discriminant analysis of principal components without prior population information. Figure S6: Functional category profiles of outlier SNPs per detection method in *Orchis italica*. Six radar plots (a–f) showing the percentage of outlier SNPs assigned to each of nine functional categories per detection method: (a) pcadapt; (b) OutFLANK; (c) 21-population pRDA, abiotic predictors; (d) 17-population pRDA, abiotic predictors; (e) 17-population pRDA, orchid mycorrhizal (OrM) community predictors; (f) 17-population pRDA, combined predictors. Functional categories were assigned based on InterPro domains and Gene Ontology terms in the *Ophrys sphegodes* annotation (Russo *et al*., 2024). *n* = number of outlier SNPs detected by that method.
