# Supplementary material for: Interplay between abiotic conditions and mycorrhizal abundance determines differentiation and potential adaptation in a Mediterranean orchid
Source: Ann Bot. 2026 Jul 23;138(5):1917–28. doi: 10.1093/aob/mcag219 (PMC13715031; doi:10.1093/aob/mcag219)
Supplement: Supplementary file 1 [file mcag219_supplementary_data.zip › Fig.S1(draft).pdf]

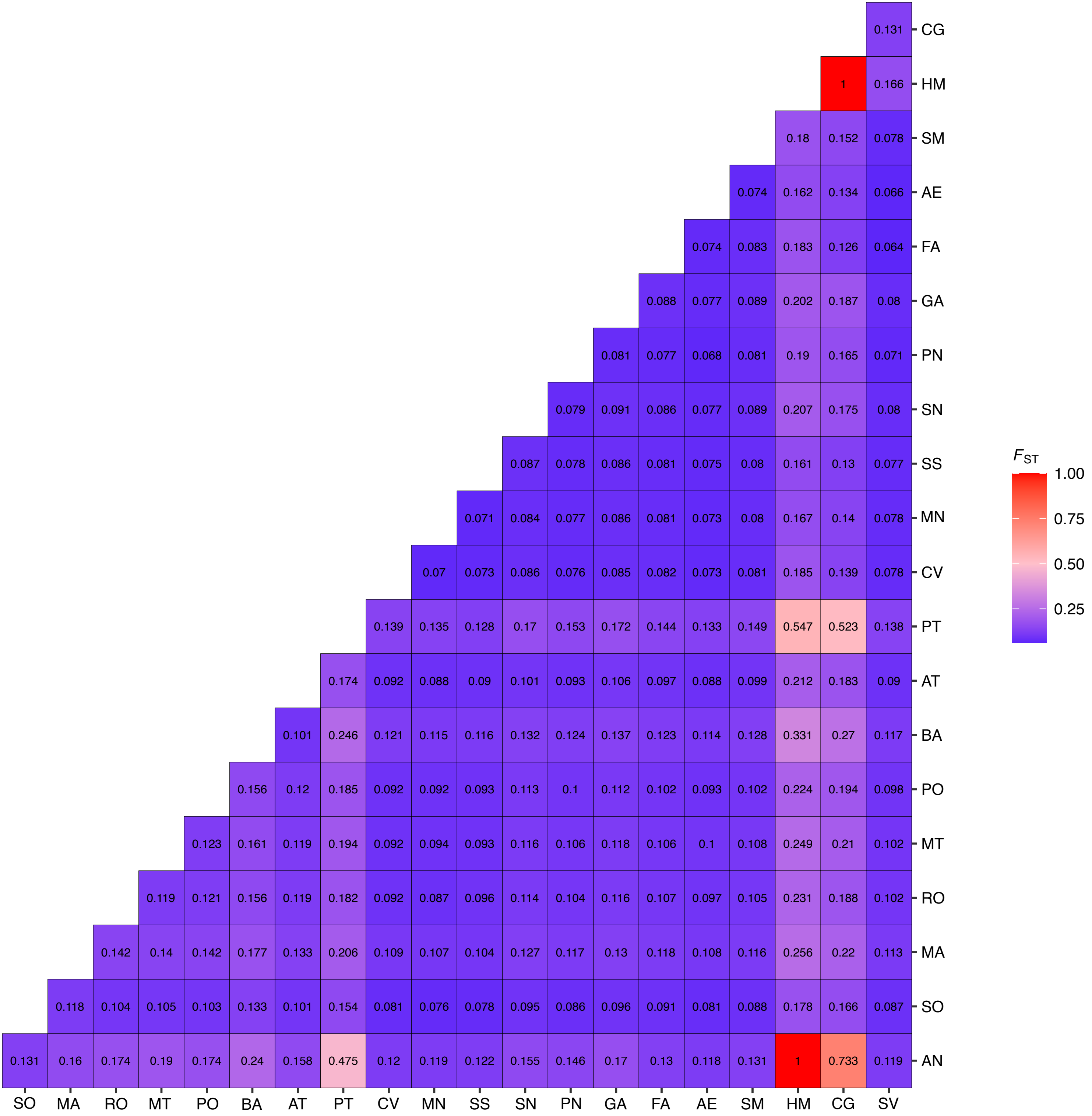

0.131 CG
1 0.166 HM
0.18 0.152 0.078 SM
0.074 0.162 0.134 0.066 AE
0.074 0.083 0.183 0.126 0.064 FA
0.088 0.077 0.089 0.202 0.187 0.08 GA
0.081 0.077 0.068 0.081 0.19 0.165 0.071 PN
0.079 0.091 0.086 0.077 0.089 0.207 0.175 0.08 SN
0.087 0.078 0.086 0.081 0.075 0.08 0.161 0.13 0.077 SS
0.071 0.084 0.077 0.086 0.081 0.073 0.08 0.167 0.14 0.078 MN
0.07 0.073 0.086 0.076 0.085 0.082 0.073 0.081 0.185 0.139 0.078 CV
0.139 0.135 0.128 0.17 0.153 0.172 0.144 0.133 0.149 0.547 0.523 0.138 PT
0.174 0.092 0.088 0.09 0.101 0.093 0.106 0.097 0.088 0.099 0.212 0.183 0.09 AT
0.101 0.246 0.121 0.115 0.116 0.132 0.124 0.137 0.123 0.114 0.128 0.331 0.27 0.117 BA
0.156 0.12 0.185 0.092 0.092 0.093 0.113 0.1 0.112 0.102 0.093 0.102 0.224 0.194 0.098 PO
0.123 0.161 0.119 0.194 0.092 0.094 0.093 0.116 0.106 0.118 0.106 0.1 0.108 0.249 0.21 0.102 MT
0.119 0.121 0.156 0.119 0.182 0.092 0.087 0.096 0.114 0.104 0.116 0.107 0.097 0.105 0.231 0.188 0.102 RO
0.142 0.14 0.142 0.177 0.133 0.206 0.109 0.107 0.104 0.127 0.117 0.13 0.118 0.108 0.116 0.256 0.22 0.113 MA
0.118 0.104 0.105 0.103 0.133 0.101 0.154 0.081 0.076 0.078 0.095 0.086 0.096 0.091 0.081 0.088 0.178 0.166 0.087 SO
0.131 0.16 0.174 0.19 0.174 0.24 0.158 0.475 0.12 0.119 0.122 0.155 0.146 0.17 0.13 0.118 0.131 1 0.733 0.119 AN
SO MA RO MT PO BA AT PT CV MN SS SN PN GA FA AE SM HM CG SV
F_{ST}
1.00
0.75
0.50
0.25
