# Supplementary material for: Interplay between abiotic conditions and mycorrhizal abundance determines differentiation and potential adaptation in a Mediterranean orchid
Source: Ann Bot. 2026 Jul 23;138(5):1917–28. doi: 10.1093/aob/mcag219 (PMC13715031; doi:10.1093/aob/mcag219)
Supplement: Supplementary file 1 [file mcag219_supplementary_data.zip › Fig.S3(draft).pdf]

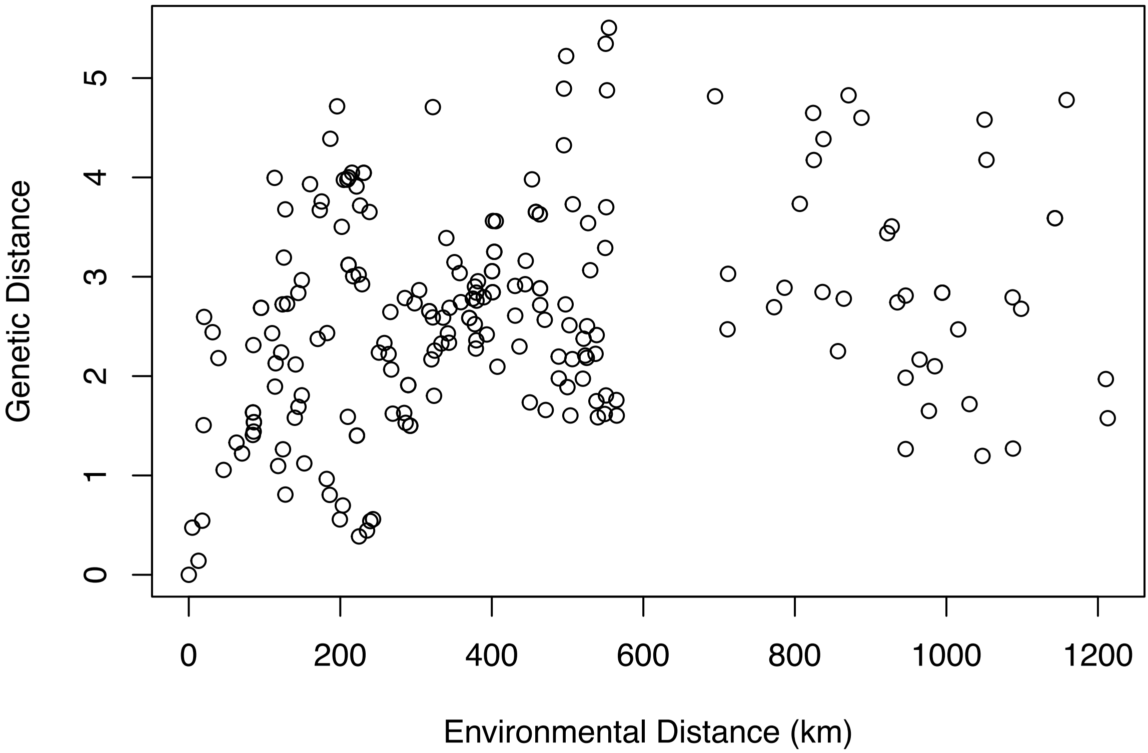
Genetic Distance
0
1
2
3
4
5
0
200
400
600
800
1000
1200
Environmental Distance (km)
