# Supplementary material for: Interplay between abiotic conditions and mycorrhizal abundance determines differentiation and potential adaptation in a Mediterranean orchid
Source: Ann Bot. 2026 Jul 23;138(5):1917–28. doi: 10.1093/aob/mcag219 (PMC13715031; doi:10.1093/aob/mcag219)
Supplement: Supplementary file 1 [file mcag219_supplementary_data.zip › Fig_S4.pdf]

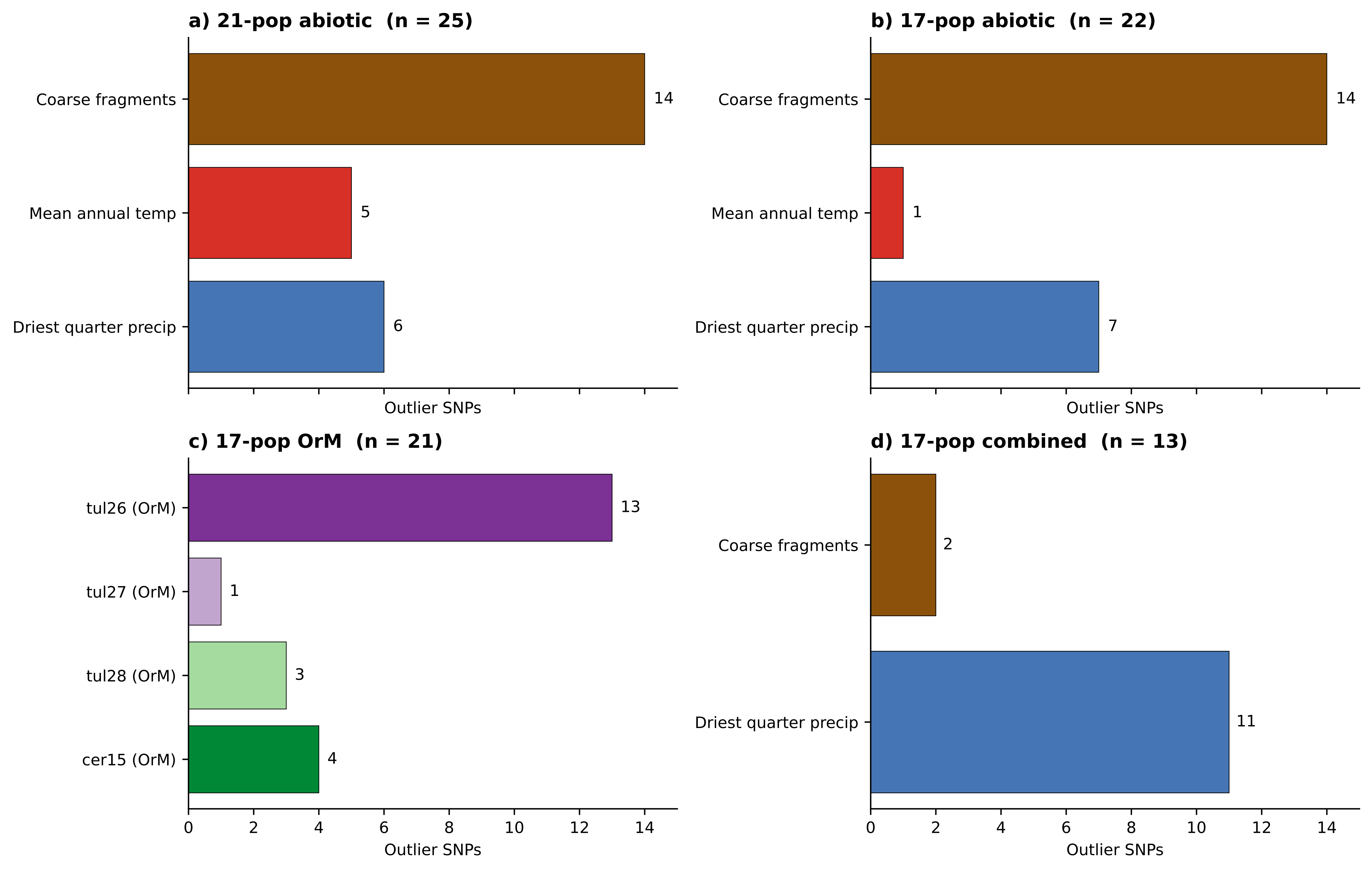

a) 21-pop abiotic (n = 25)
Coarse fragments
14
Mean annual temp
5
Driest quarter precip
6
Outlier SNPs
b) 17-pop abiotic (n = 22)
Coarse fragments
14
Mean annual temp
1
Driest quarter precip
7
Outlier SNPs
c) 17-pop OrM (n = 21)
tul26 (OrM)
13
tul27 (OrM)
1
tul28 (OrM)
3
cer15 (OrM)
4
0
2
4
6
8
10
12
14
Outlier SNPs
d) 17-pop combined (n = 13)
Coarse fragments
2
Driest quarter precip
11
0
2
4
6
8
10
12
14
Outlier SNPs
