# Supplementary material for: Interplay between abiotic conditions and mycorrhizal abundance determines differentiation and potential adaptation in a Mediterranean orchid
Source: Ann Bot. 2026 Jul 23;138(5):1917–28. doi: 10.1093/aob/mcag219 (PMC13715031; doi:10.1093/aob/mcag219)
Supplement: Supplementary file 1 [file mcag219_supplementary_data.zip › Fig_S6.pdf]

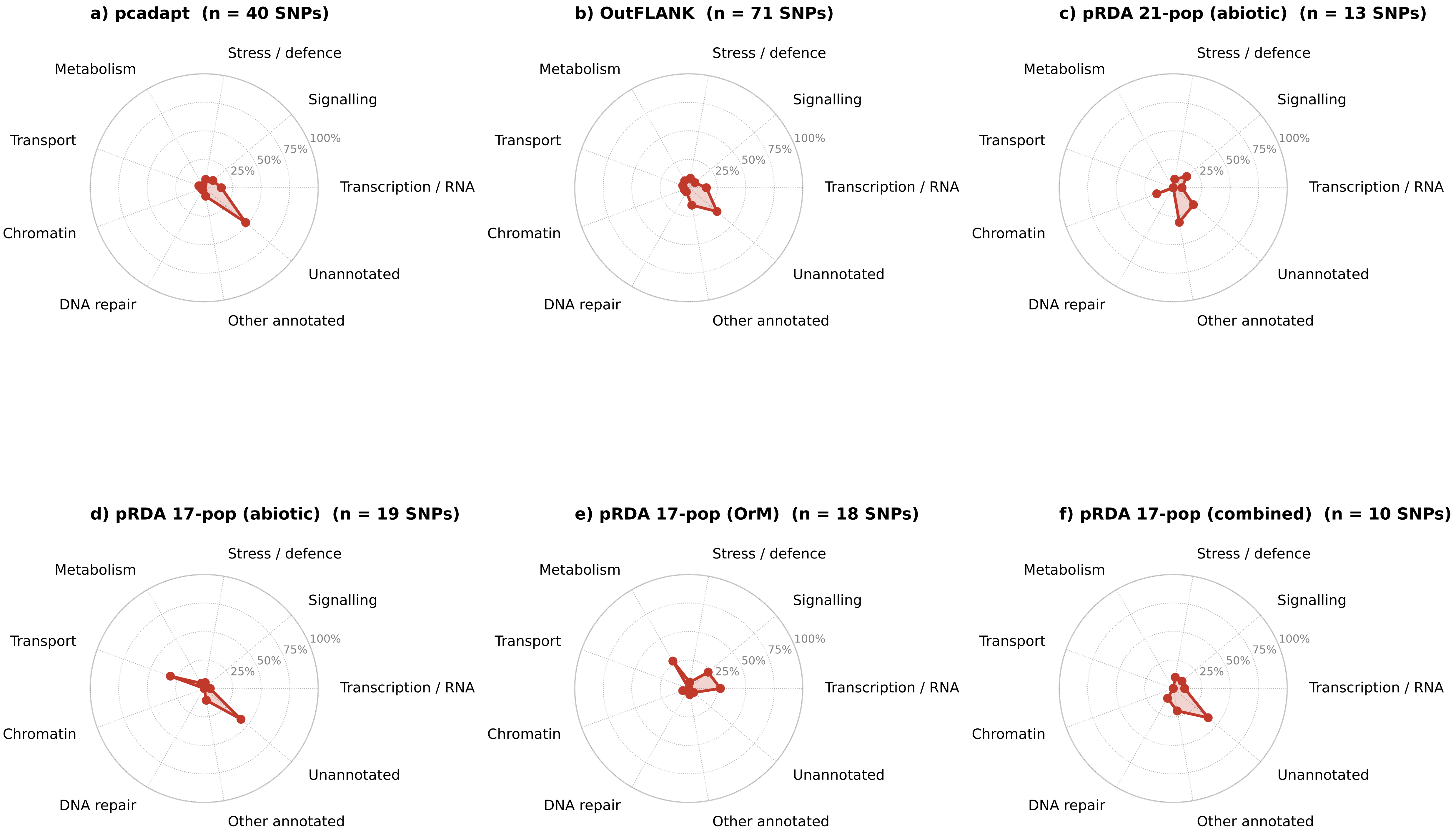

a) pcadapt (n = 40 SNPs)
b) OutFLANK (n = 71 SNPs)
c) pRDA 21-pop (abiotic) (n = 13 SNPs)
d) pRDA 17-pop (abiotic) (n = 19 SNPs)
e) pRDA 17-pop (OrM) (n = 18 SNPs)
f) pRDA 17-pop (combined) (n = 10 SNPs)
Stress / defence
Signalling
Transcription / RNA
Unannotated
Other annotated
DNA repair
Chromatin
Transport
Metabolism
25%
50%
75%
100%
